# Nicotine Reduces Human Brain Microvascular Endothelial Cell Response to *Escherichia coli* K1 Infection by Inhibiting Autophagy

**DOI:** 10.3389/fcimb.2020.00484

**Published:** 2020-09-15

**Authors:** Chao Wu, Mengzhen Yang, Rui Liu, Hanyang Hu, Lulu Ji, Xiaoli Zhang, Shenghe Huang, Lin Wang

**Affiliations:** ^1^Department of Histology and Embryology, School of Basic Medical Sciences, Wuhan University, Wuhan, China; ^2^Department of Human Anatomy, School of Basic Medical Sciences, Hubei University of Medicine, Shiyan, China; ^3^Department of Ultrasound Imaging, Zhongnan Hospital of Wuhan University, Wuhan, China; ^4^Kunming Key Laboratory of Children Infection and Immunity, Yunnan Institute of Pediatrics, Kunming Children's Hospital, Kunming, China; ^5^Department of Pediatrics, Children's Hospital Los Angeles, University of Southern California, Los Angeles, CA, United States; ^6^Hubei Provincial Key Laboratory of Developmentally Originated Disease, Wuhan, China

**Keywords:** autophagy, human brain microvascular endothelial cells, nicotine, *Escherichia coli*, neonatal meningitis

## Abstract

Studies have shown that exposure to environmental tobacco smoke can increase the risk of bacterial meningitis, and nicotine is the core component of environmental tobacco smoke. Autophagy is an important way for host cells to eliminate invasive pathogens and resist infection. *Escherichia coli* K1 strain (*E. coli* K1) is the most common Gram-negative bacterial pathogen that causes neonatal meningitis. The mechanism of nicotine promoting *E. coli* K1 to invade human brain microvascular endothelial cells (HBMECs), the main component of the blood–brain barrier, is not clear yet. Our study found that the increase of HBMEC autophagy level during *E. coli* K1 infection could decrease the survival of intracellular bacteria, while nicotine exposure could inhibit the HBMEC autophagic response of *E. coli* K1 infection by activating the NF-kappa B and PI3K/Akt/mTOR pathway. We concluded that nicotine could inhibit HBMEC autophagy upon *E. coli* K1 infection and decrease the scavenging effect on *E. coli* K1, thus promoting the occurrence and development of neonatal meningitis.

## Introduction

Neonatal bacterial meningitis (NBM) is a serious life-threatening infectious disease of the central nervous system (Heckenberg et al., 2014; Iovino et al., 2014). Although antibiotic treatment has significantly reduced the mortality rate of NBM, the morbidity rates remain unchanged; survivors often suffer from permanent neurological sequelae such as cerebral palsy, seizures, deafness, and blindness (Furyk et al., 2011; van de Beek et al., 2012; Gradstedt et al., 2013). *Escherichia coli* K1 (*E. coli K1* strain) is the most common Gram-negative bacterial pathogen causing NBM (Wang et al., 2016). High level of bacteremia is a necessary condition for meningitis (Doran et al., 2016; Kim, 2016), and nicotine (NT), the main component of tobacco smoke, can significantly enhance the invasion of human brain microvascular endothelial cells (HBMECs), which are the main component of the blood–brain barrier (Wang et al., 2003). However, the molecular mechanism of how nicotine affects the pathogenesis of *E. coli* meningitis has not been clearly understood (Bredfeldt et al., 1995; Iles et al., 2001; Chi et al., 2011b; Huang et al., 2016; Liu et al., 2019), while other microorganism-mediated autophagy play the opposite role, such as *Brucella melitensis* that can induce incomplete autophagy, which can help *B. melitensis* survive and replicate in host cells for a long time (Wang and Cheng, 1994; Siddiqi et al., 2015). In *E. coli* meningitis, α7 nAChR is critical for the activation of the NF-κB signaling pathway in HBMECs (Chen et al., 2002; Chi et al., 2011a). Under the different conditions, the continuous activation of NF-κB signaling pathway can negatively or positively regulate autophagy; it is unclear whether activation of the NF-κB signaling pathway regulates autophagy in HBMECs infected with *E. coli* K1 ADDIN EN.CITE (Koedel et al., 2000; Vallabhapurapu and Karin, 2009). There is a close correlation between NF-κB signal transduction pathway and rapamycin kinase target protein (mTOR) pathway. The activation of mTOR can inhibit autophagy, and it was regulated by multiple upstream signals, PI3K/Akt is one of the most important regulatory signal pathways (Deretic et al., 2013). Whether the NT can regulate the autophagy of *E. coli* K1–infected HBMECs through PI3K/Akt/mTOR pathway needs us to further explore.

In this study, we explored the effects of nicotine on HBMEC autophagy and the scavenging effect of HBMEC on invading intracellular bacteria, and identified this effect and clarified the role of NF-kappa B and PI3K/Akt/mTOR pathway in this process. It will help us to understand the pathogenesis of neonatal *E. coli* meningitis and provide an important theoretical basis for its prevention and treatment.

## Materials and Methods

### Cell Culture and Bacterial Infection

The human brain microvascular endothelial cell line was cultured and isolated as described in previous studies (Chi et al., 2011a, 2012). HBMEC is the main component of the blood–brain barrier. There are many tight junctions between cells, which produce high transendothelial impedance and express cell adhesion molecules. HBMEC was routinely cultured in RPMI 1640 medium, supplemented with 10% heat-inactivated fetal bovine serum, 2 mM glutamine, 1 mM sodium pyruvate, essential amino acids, vitamins, penicillin G (50 μg/ml), and streptomycin (100 μg/ml) at 37° C in 5% CO_2_. E44 is a rifampicin-resistant derivative of *E. coli* RS218 (O18:K1:H7), which was isolated from a cerebrospinal fluid from a patient with neonatal *E. coli* meningitis (Chi et al., 2011a,b) and was grown overnight in Luria–Bertani broth supplemented with rifampicin (100 μg/ml) at 37° C. For infection assays, cells were infected with E44 at a multiplicity of infection of 100 E44 to 1 HBMEC in experimental medium (1:1 mixture of M199:Ham's F-12 containing 5% heat-inactivated fetal bovine serum). To test the effects of NT on autophagy of E44-infected HBMECs, cells were preincubated with or without 5 nM αBTX for 1 h and then treated with 10^−6^ M NT for 24 h before infection. To inhibit NF-κB activation, cells were pretreated with 5 μM BAY11-7082 for 1 h before infection. In conditions where autophagy was inhibited or activated, cells were preincubated with 5 mM 3-MA or 200 nM rapamycin for 2 h before infection.

### Intracellular Bacterial Survival Assay

HBMECs were cultured in 24-well-plates and were pre-treated with or without αBTX, NT, BAY11-7082, 3-MA, and rapamycin and then infected with E44 in experimental medium at 37° C and 5% CO_2_ for 1 h. The cells were washed three times with PBS to remove free bacteria and then incubated in experimental medium containing gentamicin (100 μg/ml) for 1 h to kill extracellular bacteria. Half of the wells in each treatment group were washed three times with PBS and lysed with 0.5% Triton X-100, and then intracellular bacteria were counted by plating serial dilutions of the lysates on Luria–Bertani solid medium plates to enumerate CFU_t1_. The remaining wells were subjected to further incubation for 1 h, and intracellular bacteria were enumerated as described previously (CFU_t2_). Intracellular survival (%) was calculated as CFU_t2_/CFU_t1_ × 100%.

### Western Blotting

Protein samples were boiled for 5 min in 5× SDS-PAGE loading buffer and loaded onto 10–15% SDS polyacrylamide gels. Proteins were transferred to PVDF membranes, and membranes were blocked for 2 h in Tris-buffered saline (pH 7.6) containing 0.1% Tween 20 with 5% non-fat dry milk and then incubated at 4° C overnight with the following primary antibodies: anti-LC3B (1:2,000; Sigma-Aldrich), anti-P62 (1:5,000; Proteintech), anti-P65 (1:1,000; Cell Signaling Technology), anti-phospho-P65 (1:1,000; Cell Signaling Technology), anti-ICAM-1 (1:200; Santa Cruz Biotechnology), anti-PI3K (1:1,000; Cell Signaling Technology), anti-phospho-PI3K (1:1,000; Cell Signaling Technology), anti-Akt (1:1,000; Cell Signaling Technology), anti-phospho-Akt (1:1,000; Cell Signaling Technology), anti-phospho-p70S6K (1:1,000; Cell Signaling Technology), or anti-GAPDH(1:50,000; Proteintech). Blots were washed and then incubated with the HRP-conjugated secondary antibody (1:10,000; Proteintech), and blots were revealed using ECL. ImageJ software was used for the semi-quantification of protein expression.

### Transmission Electron Microscopy

Samples were fixed with 2.5% glutaraldehyde and incubated with 1% osmium tetroxide in 0.1 M sodium cacodylate buffer and embedded in epoxy resin. Sections were cut at a nominal thickness of 80 nm and stained with uranyl acetate and lead citrate. Images were recorded using a Hitachi-7700 transmission electron microscope (Hitachi Limited, Tokyo, Japan).

### Confocal Laser Scanning Microscopy

HBMECs were transfected with 100 μl OptiMEM medium (Gibco/BRL) containing 1% Lipofectamine 2000 (Invitrogen) and 1 μg of mCherry-GFP LC3B plasmid. After 6 h, the medium was changed to normal medium, and after transfection for 48 h, the transfected cells were treated with or without NT, E44, and BAY11-7082. Transfected cells were examined by confocal microscopy.

### Statistical Analysis

All experiments were performed in triplicate and repeated at least three times. Statistically significant differences between groups were determined using two-tailed one-way ANOVA, followed by a Student–Newman–Keuls test or Student *t*-test. *P* < 0.05 was considered statistically significant.

## Results

### Nicotine Inhibits Autophagy in *E. coli* K1–Infected HBMEC

**Figure 1:**
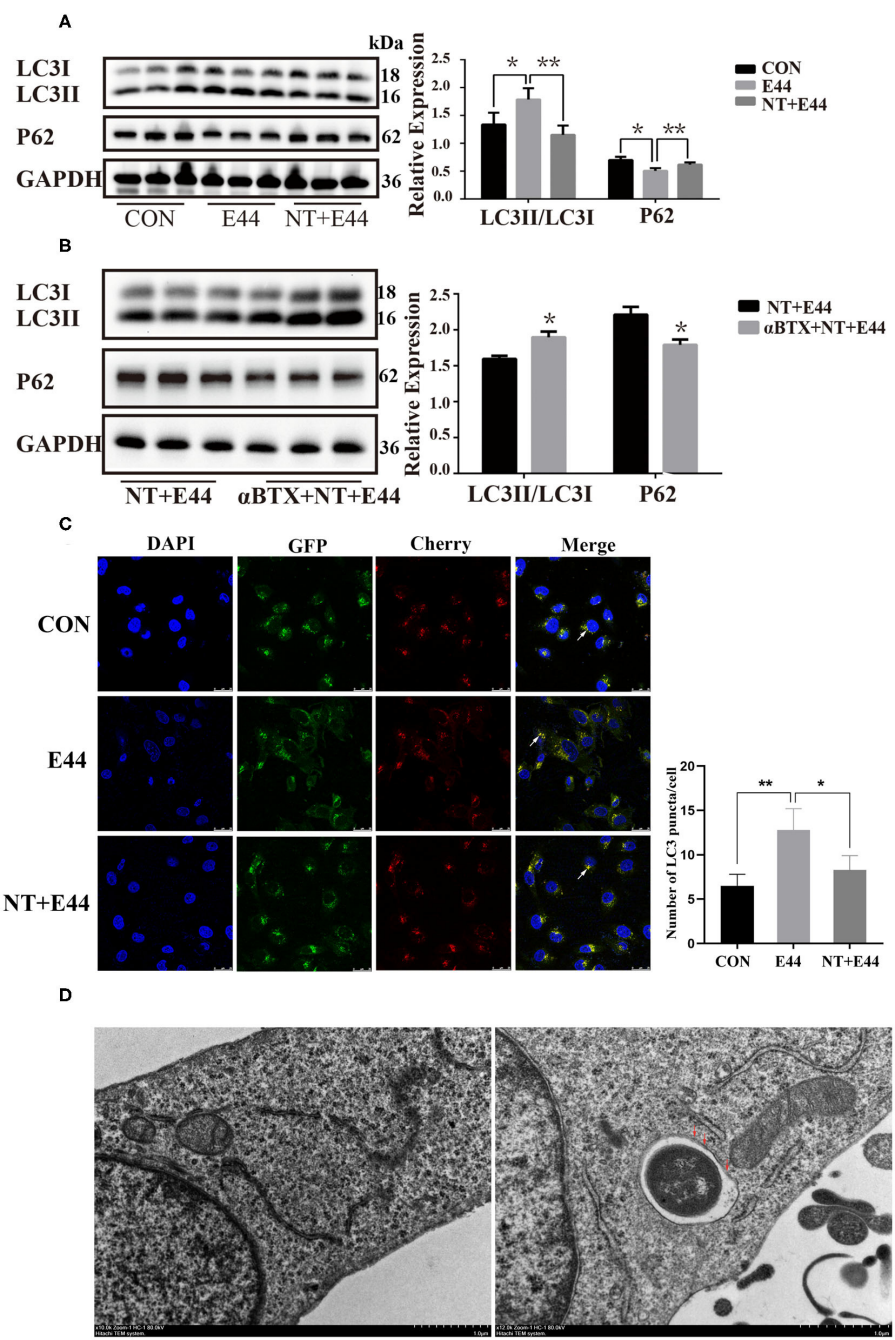
Effects of nicotine exposure on autophagy in E44 infected HBMECs. **(A,B)** HBMECs were subjected to the following treatments: without any treatment (CON); infection with E44 for 2 h (E44); incubation with NT for 24 h and then infection with E44 for 2 h (NT+E44); treatment with αBTX for 1 h, then incubation with NT for 24 h, and finally infection with E44 for 2 h (αBTX+NT+E44). Autophagy-related proteins LC3 and P62 were analyzed by Western blotting (left). Quantitative analysis was performed using ImageJ software to determine p62/GAPDH and LC3II/LC3I ratios (right). **(C)** Representative confocal microscopic images of HBMECs transfected with mCherry-GFP-LC3 (the white arrows indicate autophagosomes). Scale bar, 25 μm. **(D)** Two hours after E44 infection of HBMECs, the intracellular structure was observed by transmission electron microscopy. Extracellular E44 invaded HBMECs. E44 wrapped in a double membrane (the red arrows indicate the double membrane structure inside the cell). Scale bar, 1.0 μm. Data are means ± SEM of three independent experiments conducted in triplicate. GAPDH, glyceraldehyde 3-phosphate dehydrogenase; DAPI, 40,6-diamidino-2phenylindole. *P < 0.05 and **P < 0.01.

To determine whether *E. coli* K1–infected HBMEC acted directly on autophagy, we used HBMECs exposed to different time of E44 in the culture medium. We assessed levels of autophagy-associated proteins. LC3 is ubiquitous in mammalian cells and is the most characteristic core autophagy-related protein. Upon initiation of autophagy, LC3I in the cytoplasm is converted to LC3II, is covalently linked to phosphatidylethanolamine and bound to the autophagosome membrane (Tanida et al., 2008). Subsequently, the p62/SQSTM1 protein transports cargo such as ubiquitinated protein aggregates, ubiquitin-labeled bacteria, etc. into the autophagosome by interacting with the LC3 protein (Pankiv et al., 2007). Finally, p62/SQSTM1 proteins are degraded by autolysosome (Bjorkoy et al., 2005). Therefore, LC3II and p62/SQSTM1 proteins can be used as markers for detecting autophagy. To investigate autophagy level of HBMECs in the context of *E. coli* K1 infection, HBMECs were infected with *E. coli* strain E44 for different amounts of time, and the results showed the expression of LC3II in E44-infected HBMECs in a time-dependent manner, with peak conversion at 2 h (Supplementary Figure 1). Next, HBMECs were exposed to low doses of NT (10 μM) for 24 h and then infected with E44 for 2 h. Western blotting results showed that NT significantly blocked the expression of LC3II and promoted the expression of p62 (Figure 1A). To conclusively determine whether nicotine inhibits E44-infected HBMEC autophagy, 5 nM α-bungarotoxin (αBTX, a nicotinic acetylcholine receptor antagonist) was added for 1 h before treatment with NT. Western blotting results indicated that αBTX significantly blocked the effect of NT on autophagy-related proteins p62 and LC3 in E44-infected HBMECs (Figure 1B). We further transfected HBMECs with mCherry-GFP-LC3B plasmid. In the early stages, the autophagosomes were double-labeled by Cherry and GFP to appear yellow. In the later stage, the autophagosomes fused with lysosomes, and the acidic lysosomal environment quenched GFP, appearing red. We found that E44 infection increased the abundance of yellow and red puncta compared with that in the uninfected group, and the abundance of both puncta in the NT pretreatment group decreased (Figure 1C). Together, these results suggest that E44 infection promoted autophagy in HBMECs and that NT significantly blocked E44-induced autophagy. Our results have shown that autophagy of HBMECs is significantly activated when cells were infected with E44 for 2 h, it is unclear whether E44 resides in the double membrane structures typical of autophagosomes. Using transmission electron microscopy (TEM) analysis, we found that after 2 h of infection, E44 was found in double membrane structures of autophagosomes (Figure 1D).

### Nicotine Promotes NF-κB Activation to Inhibit Autophagy

**Figure 2:**
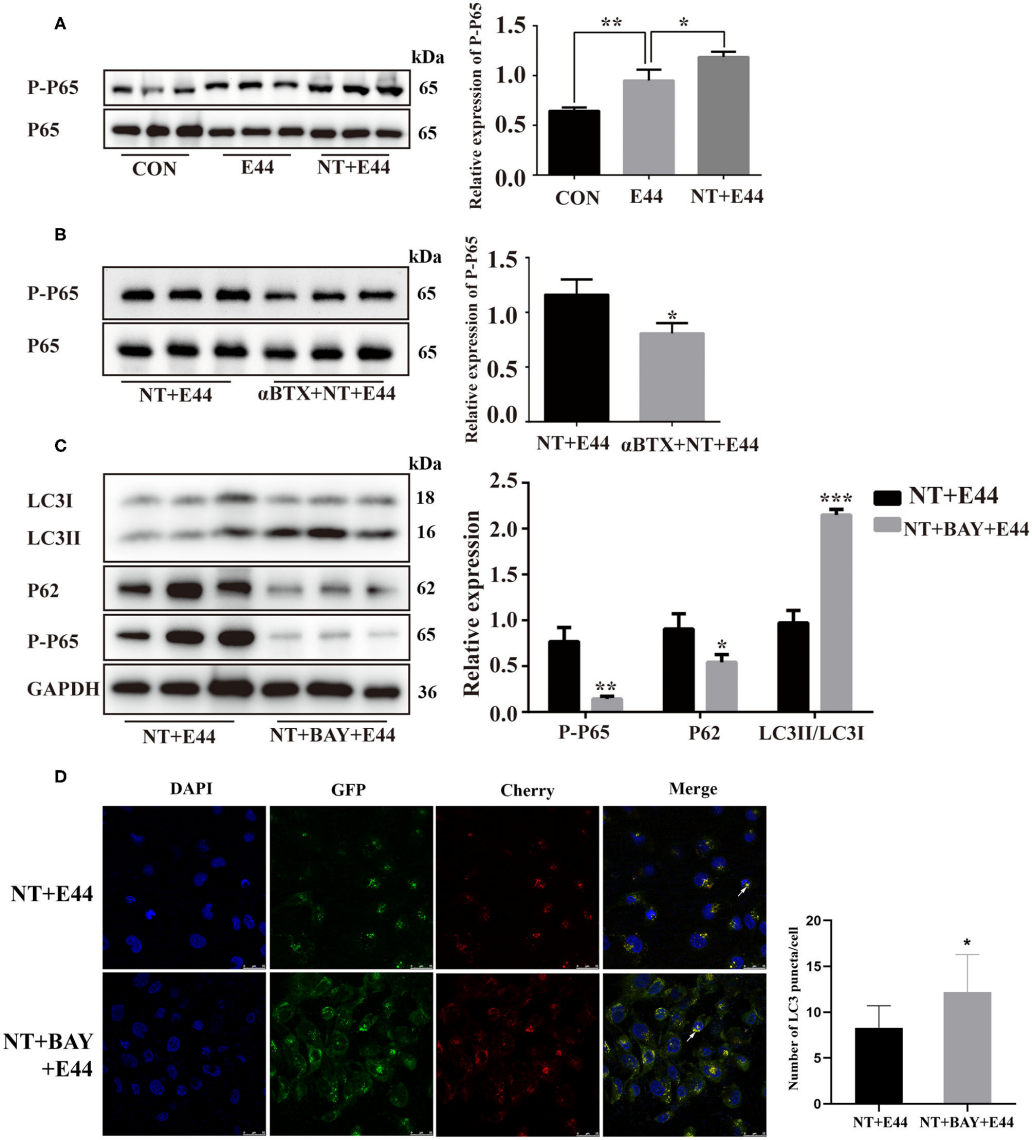
Effect of NT on NF-κB activation in E44-infected HBMECs. **(A,B)** HBMECs were subjected to the following treatments: without any treatment (CON); infection with E44 for 2 h (E44); incubation with NT for 24 h and then infection with E44 for 2 h (NT+E44); treatment with αBTX for 1 h, then incubation with NT for 24 h, and finally infection with E44 for 2 h (αBTX+NT+E44). Phosphorylated p65 and total P65 were analyzed by Western blotting (left). Quantitative analysis was performed using ImageJ software to determine P-P65/P65 ratios (right). **(C)** HBMECs were subjected to the following treatments: incubation with NT for 24 h and then infection with E44 for 2 h (NT+E44); incubation with NT for 24 h and BAY11-7082 (BAY) for 1 h and then infection with E44 for 2 h (NT+BAY+E44). Quantitative analysis was performed using ImageJ software to determine P-P65/GAPDH, p62/GAPDH, and LC3II/LC3I ratios (right). **(D)** Representative confocal microscopic images of HBMECs transfected with mCherry-GFP-LC3 (the white arrows indicate autophagosomes). Scale bar, 25 μm. Data are means ± SEM of 3 independent experiments conducted in triplicate. GAPDH, glyceraldehyde 3-phosphate dehydrogenase; DAPI, 40,6-diamidino-2phenylindole. *P < 0.05; **P < 0.01; and ***P < 0.001.

We next assessed whether NT promotes NF-κB activation to inhibit autophagy on *E. coli* K1–infected HBMEC. Previous studies have shown that α7 nAChR plays an important role in the activation of NF-κB during the pathogenesis of *E. coli* meningitis (Huang et al., 2016). However, it is unclear whether NT regulates the activation of NF-κB in E44-infected HBMECs. We pretreated HBMECs with or without NT for 24 h and then infected them with E44 for 2 h. Studies have confirmed that phosphorylation of the Ser-536 site of the p65 subunit can serve as a marker of NF-κB activation (Ahmed et al., 2014). Western blotting results showed that NT significantly promoted the expression level of p-p65 (Figure 2A). Next, αBTX (5 nM) was added for 1 h before treatment with NT, and the protein level of P-P65 decreased significantly (Figure 2B). To further investigate whether NF-κB activation affects autophagy, we inhibited NF-κB with BAY11-7082 (5 μM), and the results showed that the protein level of LC3II was significantly increased and that p62 levels were significantly decreased (Figure 2C). We transfected HBMECs with the mCherry-GFP-LC3B plasmid and found that the inhibition of NF-κB activation significantly increased the abundance of yellow and red puncta (Figure 2D). Together, these results suggest that NT promotes NF-κB activation during E44 infection and inhibits autophagy.

### Nicotine Promotes Activation of the Autophagy-Related PI3K/Akt/mTOR Signaling Pathway

**Figure 3:**
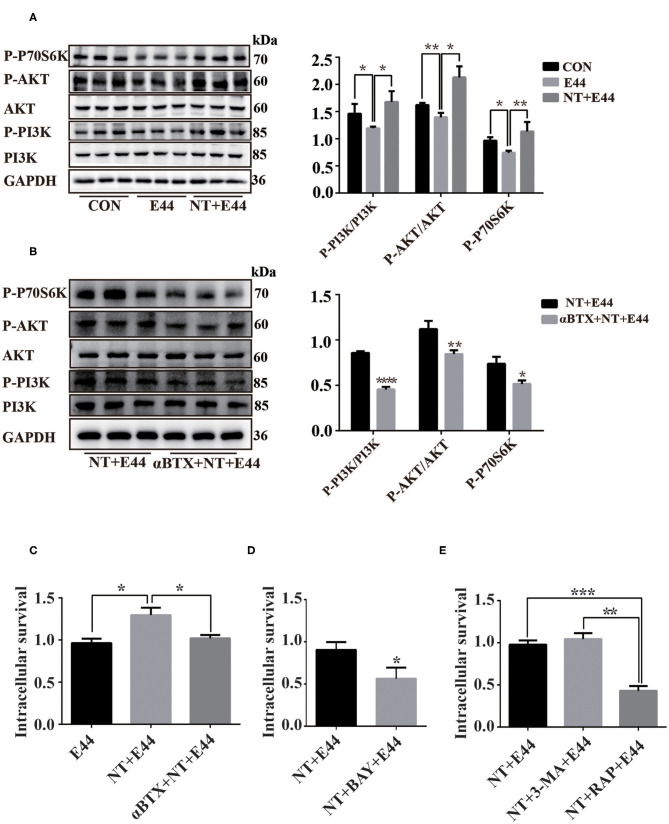
The effect of NT on activation of PI3K/AKT/mTOR signaling pathway in E44-infected HBMECs. **(A,B)** HBMECs were subjected to the following treatments: without any treatment (CON); infection with E44 for 2 h (E44); incubation with NT for 24 h and then infection with E44 for 2 h (NT+E44); treatment with αBTX for 1 h, then incubation with NT for 24 h, and finally infection with E44 for 2 h (αBTX+NT+E44). P-PI3K, P-Akt, P-P70S6K, PI3K, Akt were analyzed by Western blotting (left). Quantitative analysis was performed using ImageJ software to determine P-PI3K/PI3K, P-Akt/Akt, and P-P70S6K/GAPDH ratios (right). **(C)** The effect of nicotine exposure on E44 survival in HBMEC. **(D)** The effect of inhibition of NF-κB activation by BAY11-7082 (BAY) on the survival of E44 in HBEMC. **(E)** The effect of autophagy on the survival of E44 in HBMEC. Data are means ± SEM of three independent experiments conducted in triplicate. GAPDH, glyceraldehyde 3-phosphate dehydrogenase. *P < 0.05; **P < 0.01; and ***P < 0.001.

**Figure 4:**
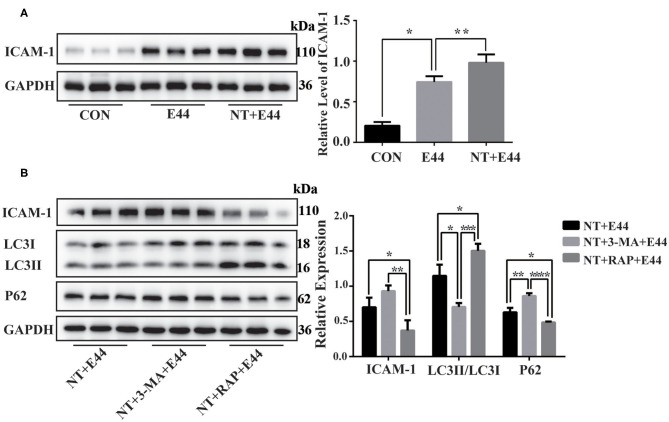
The expression of ICAM-1 in E44-infected HBMEC. **(A)** Effect of nicotine on ICAM-1 expression in E44-infected HBMEC. HBMECs were subjected to the following treatments: without any treatment (CON); infection with E44 for 2 h (E44); incubation with NT for 24 h and then infection with E44 for 2 h (NT+E44); treatment with αBTX for 1 h, then incubation with NT for 24 h, and finally infection with E44 for 2 h (αBTX+NT+E44). ICAM-1 were analyzed by Western blotting (left). Quantitative analysis was performed using ImageJ software to determine ICAM-1/GAPDH ratios (right). **(B)** Effect of autophagy on ICAM-1 expression in E44-infected HBMEC. HBMECs were subjected to the following treatments: incubation with NT for 24 h and then infection with E44 for 2 h (NT+E44); incubation with NT for 24 h and 3-MA for 2 h and then infection with E44 for 2 h (NT+3-MA+E44); incubation with NT for 24 h and RAP for 2 h and then infection with E44 for 2 h (NT+RAP+E44). ICAM-1, LC3, and P62 were analyzed by Western blotting (left). Quantitative analysis was performed using ImageJ software to determine ICAM-1/GAPDH, p62/GAPDH, and LC3II/LC3I ratios (right). Data are means ± SEM of three independent experiments conducted in triplicate. GAPDH, glyceraldehyde 3-phosphate dehydrogenase. *P < 0.05; **P < 0.01; ***P < 0.001; and ****P < 0.0001.

We began our investigation of the drivers of autophagy-related PI3K/Akt/mTOR signaling pathway in *E. coli* K1–infected HBMEC by hypothesizing that the NT playing a detrimental role in HBMECs' defense against *E. coli* meningitis may act as an infection-associated regulator of autophagy. mTOR is a serine/threonine kinase composed of two signal complexes, mTORC1 and mTORC2, and mTORC1 activation inhibits autophagy (Jung et al., 2009). Activation of mTORC1 directly promotes phosphorylation of the downstream target ribosomal protein S6 kinase (P70S6K), and the phosphorylation level of P70S6K can reflect the activation of mTORC1 (Laplante and Sabatini, 2012). Previous studies have shown that *E. coli* K1 activates PI3K/Akt in a time-dependent manner, with peak at 10–15 min of infection (Sukumaran et al., 2003; Zhao et al., 2010), and NT significantly promotes activation of Akt in HBMEC infected with *E. coli* K1 for 20 min (Chen et al., 2002). Our results have confirmed that E44 infection for 2 h significantly promoted autophagy of HBMEC, but was inhibited under nicotine exposure. It is unclear whether the PI3K/Akt/mTOR signaling pathway is involved in the regulation of autophagy in E44-infected HBMECs. Therefore, the present study aimed to further examine whether NT may affect E44-infected HBMEC autophagy through the PI3K/Akt/mTOR pathway. The levels of PI3K, P-PI3K, Akt, P-Akt, and P-P70S6K were determined by western blotting. The results showed that the PI3K/Akt/mTOR pathway was inhibited by 2 h of E44 infection of HBMECs, and NT pretreatment significantly activated the PI3K/Akt/mTOR pathway (Figure 3A). Next, αBTX was used to block NT binding to α7 nAChR, and PI3K/Akt/mTOR pathway activation was blocked (Figure 3B). To further investigate whether NT affected E44-infected HBMEC autophagy via the PI3K/Akt/mTOR signaling pathway, we inhibited mTOR with rapamycin, and the results showed that the protein level of LC3II was significantly increased and that p62 levels were significantly decreased (Figure 4B). These results indicate that NT significantly promotes activation of the autophagy-related PI3K/Akt/mTOR signaling pathway in E44-infected HBMECs.

### The Autophagy Pathway Limits Intracellular E44 Survival

Although nicotine inhibits autophagy in E44-infected HBMECs, it is unclear whether inhibition of autophagy affects intracellular E44 survival. Thus, we tested the survival rate of E44 in HBMECs. Compared with in the E44 treatment alone group, the survival rate of intracellular E44 was significantly increased in the NT + E44 treatment group, and the survival rate of E44 was significantly decreased after αBTX blocked NT binding to α7 nAChR (Figure 3C). Compared with in the NT + E44 treatment group, the survival rate of E44 was significantly decreased in the BAY11-7082 treatment group (Figure 3D). To investigate the effect of autophagy on intracellular E44 survival, we activated and inhibited autophagy with the autophagy inducer rapamycin (200 nM) and the autophagy inhibitor 3-MA (5 mM) (Figure 4B), respectively, and rapamycin significantly reduced the intracellular E44 survival rate (Figure 3E). The aforementioned results indicate that autophagy facilitates the clearance of intracellular *E. coli* K1 by HBMECs. NT promotes the survival of E44 in HBMECs by inhibiting autophagy.

### Induction of Autophagy Inhibits the Expression of ICAM-1

Finally, we sought to determine whether autophagy inhibits the expression of ICAM-1. The adhesion molecule ICAM-1 plays an important role in the migration of PMNs.

Western blotting results showed that NT promotes the expression of ICAM-1 in E44-infected HBMECs (Figure 4A). To further investigate the effect of autophagy on ICAM-1 expression, we used the autophagy inhibitor 3-MA and the autophagy inducer rapamycin to inhibit and activate autophagy, respectively, in HBMECs. Western blotting results showed that ICAM-1 expression was downregulated after treatment with the autophagy inducer rapamycin (Figure 4B). Together, these results suggest that NT promotes the activation of NF-κB in E44-infected HBMECs and promotes the expression of ICAM-1, while autophagy inhibition may be beneficial for ICAM-1 expression.

## Discussion

NT is a major component of environmental tobacco smoke, with multiple damaging effects on blood vessels, immunity, and the nervous system. Currently, studies have shown that nicotine promotes the pathogenesis of *E. coli* meningitis through the cholinergic α7 nAChR pathway. Autophagy is a fundamental eukaryotic pathway that balances the beneficial and adverse effects of immunity and inflammation and thereby may prevent infectious, autoimmune, and inflammatory diseases (Levine et al., 2011; Deretic et al., 2013). However, there is lack of information about whether and how nicotine affects the autophagy of *E. coli* K1–infected HBMECs. In this study, we confirmed the effect of nicotine on autophagy in E44-infected HBMECs. Furthermore, the function of autophagy in *E. coli* K1–infected HBMECs and the underlying molecular mechanism of NT-mediated regulation of these effects were elucidated.

Bacterial pathogens invade the blood–brain barrier endothelium, and traversing the barrier is a critical step during the development of neonatal *E. coli* meningitis. Previous studies have confirmed that autophagy is essential for HBMECs to clear Group B Streptococcus, the most common Gram-positive bacterial pathogen causing neonatal meningitis (Cutting et al., 2014). In this study, our results provide new evidence that autophagy is activated in *E. coli* K1–infected HBMECs at early time points and can limit intracellular bacterial survival, whereas chronic nicotine exposure significantly inhibits intracellular antibacterial autophagy formation.

MTOR is a serine/threonine kinase composed of two signal complexes, mTORC1 and mTORC2, and mTORC1 activation inhibits autophagy (Jung et al., 2009). The activation of mTOR is regulated by multiple upstream signals, and PI3K/Akt is one of the most important regulatory signaling pathways (Manning and Cantley, 2007). Previous studies have shown that *E. coli* K1 activates PI3K/Akt in a time-dependent manner, with peak at 10–15 min of infection (Sukumaran et al., 2003; Zhao et al., 2010), and NT significantly promotes activation of Akt in HBMEC infected with *E. coli* K1 for 20 min (Chen et al., 2002). In this study, HBMECs were infected with *E. coli* K1 for 2 h and found that activation of the PI3K/Akt/mTOR signaling pathway was inhibited and NT significantly reversed the above inhibition.

The NF-κB signaling pathway is activated during bacterial meningitis, which plays an important role in mediating *E. coli* K1 invasion of HBMECs and PMN migration across the blood–brain barrier (Chi et al., 2012); blocking NF-κB signaling can suppress bacterial meningitis (Koedel et al., 2000; Chi et al., 2012). Recent findings have shown that phosphorylation of the p65 subunit at Ser-536 is a novel mechanism of NF-κB transcriptional activation (Douillette et al., 2006; Nicholas et al., 2007; Ahmed et al., 2014). In this study, we showed that chronic NT exposure significantly promoted the phosphorylation of p65 Ser-536. Next, we inhibited the activation of NF-κB and found that the level of autophagy increased significantly and that the intracellular survival rate of *E. coli* K1 decreased. Activation of NF-κB promotes the expression of ICAM-1, which is critical for PMN binding to HBMECs and migration. Our results show that promoting autophagy significantly inhibits ICAM-1 protein expression.

Taken together, these findings suggest that NT promotes the activation of the NF-κB and PI3K/Akt/mTOR signaling pathway in *E. coli* K1–infected HBMECs, which inhibits autophagy, promotes the survival of intracellular pathogens, and facilitates the expression of ICAM-1.

## Data Availability Statement

All datasets generated for this study are included in the article/Supplementary Material.

## Author Contributions

CW and MY: conceived the project, carried out the experiments, and drafted the article. RL and HH: study design, analysis of the data, and critical revision of the article. LJ and XZ: data collection and critical revision of the article. SH: revision of the article. LW: study design and critical revision of the article. All authors contributed to the article and approved the submitted version.

## Conflict of Interest

The authors declare that the research was conducted in the absence of any commercial or financial relationships that could be construed as a potential conflict of interest.
