# Supplementary material for: Nicotine Reduces Human Brain Microvascular Endothelial Cell Response to Escherichia coli K1 Infection by Inhibiting Autophagy
Source: Front Cell Infect Microbiol. 2020 Sep 15;10:484. doi: 10.3389/fcimb.2020.00484 (PMC7522313; doi:10.3389/fcimb.2020.00484)
Supplement: Supplementary file 1 [file Data_Sheet_1.doc]

**Supplementary**

**
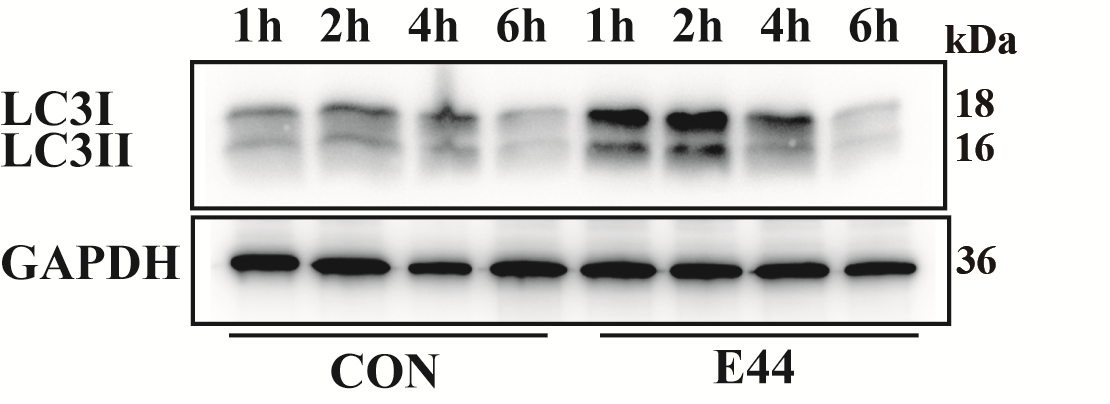
**

**Figure S1. Effects of E44 infection on autophagy in HBMEC.** HBMECs were treated with 1×10^7^ CFU/ml E44 for different amounts of time (1h, 2h, 4h, 6h), and the expression of LC3II protein was detected by western blot.
